# Emerging Oculomic Signatures: Linking Thickness of Entire Retinal Layers with Plasma Biomarkers in Preclinical Alzheimer’s Disease

**DOI:** 10.3390/jcm15010275

**Published:** 2025-12-30

**Authors:** Ibrahim Abboud, Emily Xu, Sophia Xu, Aya Alhasany, Ziyuan Wang, Xiaomeng Wu, Natalie Astraea, Fei Jiang, Zhihong Jewel Hu, Jane W. Chan

**Affiliations:** 1 Doheny Image Analysis Laboratory, Doheny Eye Institute, 150 N. Orange Grove Blvd., Pasadena, CA 91103, USA; iabboud@doheny.org (I.A.);; 2 Analytical Biochemistry Core Laboratory, Huntington Medical Research Institute, 686 S. Fair Oaks Avenue, Pasadena, CA 91105, USA; xiaomeng.wu@hmri.org (X.W.); natalie.astraea@hmri.org (N.A.); 3 Department of Epidemiology and Biostatistics, University of California, San Francisco, 50 16th Street, #2538, San Francisco, CA 94143, USA; 4 Department of Ophthalmology, University of California, Los Angeles, 100 Stein Plaza Driveway, Los Angeles, CA 90095, USA

**Keywords:** pre-Alzheimer’s disease, photoreceptor inner segment thinning, inner retina, outer retina, choroid, non-invasive, non-contact, surrogate markers, plasma p-tau217, plasma p-tau181, plasma neurofilament light chain, plasma glial fibrillary acidic protein, plasma Aβ42/40 ratio

## Abstract

**Background/Objectives**: Alzheimer’s disease (AD) is the leading cause of dementia, which is an inevitable consequence of aging. Early detection of AD, or detection during the pre-AD stage, is beneficial, as it enables timely intervention to reduce modifiable risk factors, which may help prevent or delay the progression to dementia. On the one hand, plasma biomarkers have demonstrated great promise in predicting cognitive decline. On the other hand, in recent years, ocular imaging features, particularly the thickness of retinal layers measured by spectral-domain optical coherence tomography (SD-OCT), are emerging as possible non-invasive, non-contact surrogate markers for early detection and monitoring of neurodegeneration. This pilot study aims to identify retinal layer thickness changes across the entire retina linked to plasma AD biomarkers in cognitively healthy (CH) elderly individuals at risk for AD. **Methods**: Eleven CH individuals (20 eyes total) were classified in the pre-AD stage by plasma β-amyloid (Aβ)42/40 ratio < 0.10 and underwent SD-OCT. A deep-learning-derived automated algorithm was used to segment retinal layers on OCT (with manual correction when needed). Multiple layer thicknesses throughout the entire retina (including the inner retina, the outer retina, and the choroid) were measured in the inner ring (1–3 mm) and outer ring (3–6 mm) of the Early Treatment Diabetic Retinopathy Study (ETDRS). Relationships between retinal layers and plasma biomarkers were analyzed by ridge regression/bootstrapping. **Results**: Results showed that photoreceptor inner segment (PR-IS) thinning had the largest size effect with neurofilament light chain. Additional findings revealed thinning or thickening of the other retinal layers in association with increasing levels of glial fibrillary acidic protein and phosphorylated tau at threonine 181 and 217 (p-tau181 and p-tau217). **Conclusions**: This pilot study suggests that retinal layer-specific signatures exist, with PR-IS thinning as the largest effect, indicating neurodegeneration in pre-AD. Further research is needed to confirm the findings of this pilot study using larger longitudinal pre-AD cohorts and comparative analyses with healthy aging adults.

## 1. Introduction

Alzheimer’s disease (AD), the most prevalent etiology of dementia, is strongly linked to the aging process. Detecting AD at an early or preclinical phase is critical, as it creates opportunities for early intervention aimed at modifying risk factors that could delay or mitigate progression to dementia. Although plasma-based biomarkers have shown considerable utility in anticipating cognitive decline, growing evidence supports the use of ocular imaging as an alternative approach. Advances in spectral-domain optical coherence tomography (SD-OCT) have highlighted retinal layer thickness as a potential noninvasive, contact-free surrogate marker for the early identification and monitoring of neurodegenerative processes.

The temporal relationships for pre-AD among blood-based biomarkers, such as plasma β-amyloid (Aβ)42/Aβ40, neurofilament light chain (NfL), glial fibrillary acidic protein (GFAP), and phosphorylated tau (p-tau) variants, can provide valuable insights into disease progression and support early diagnosis. Compared to other blood-based biomarkers, GFAP has been linked to clinical AD incidence more than a decade before diagnosis, approximately 9–17 years prior [1]. Subtle longitudinal changes in NfL have been observed 16 years before the onset of cognitive decline, marking its early change point [2]. Plasma biomarkers such as phosphorylated tau at threonine p-tau epitopes, especially phosphorylated tau at threonine 127 (p-tau217), demonstrate high accuracy in detecting amyloid and tau pathology and in predicting cognitive decline, often surpassing other plasma biomarkers like NfL [3]. In clinical settings, p-tau217 is used to confirm AD pathology and prognosis [4]. Therefore, these plasma biomarkers together can significantly enhance predictive capabilities.

Phosphorylated tau levels, such as p-tau217 and phosphorylated tau at threonine 181 (p-tau181), indicate tau pathology and neurofibrillary tangle formation in Alzheimer’s disease. These markers increase notably in the preclinical stages, potentially in response to Aβ42 exposure. P-tau181 and p-tau217 rise even with subtle Aβ pathology changes, helping to differentiate Aβ-positive from Aβ-negative cognitively unimpaired individuals [5]. For example, GFAP and NfL, when combined with established risk factors like age, sex, and ApoE ξ4, show strong discriminatory power in distinguishing amyloid-positive from amyloid-negative individuals, facilitating more precise diagnostic pathways for AD [6]. While p-tau biomarkers, such as p-tau217, are often favored for their specificity in detecting AD pathology, GFAP and NfL remain valuable over a longer time period for monitoring disease progression and cognitive decline in AD [7,8].

OCT is a non-invasive imaging approach that captures 3D retinas without contact with the eye surface and has no radiation. Several studies using OCT have measured the inner retinal layer thicknesses, including the retinal nerve fiber layer, ganglion cell layer, and inner plexiform layer, as potential surrogate markers for neurodegenerative changes in the brain during the preclinical AD stage [9,10,11]. Ganglion cell layer thinning has been shown to be correlated with CSF p-tau-181, total tau, and hippocampal volume in CH individuals [12,13,14,15], but more complete retinal layers across the entire retina have not yet been examined together with plasma AD biomarkers during the earliest stages of pre-AD. Our group’s deep-learning-derived, graph-based automated algorithm (currently the only method capable of segmenting thirteen retinal layers across the entire retina) offers a valuable opportunity to quantify the retina more comprehensively [16,17,18].

Thus, in this cross-sectional pilot study, we aimed to examine the layer thicknesses across the entire retina, including the inner retina, the outer retina, and the choroid, in relation to plasma Aβ42/40 ratio, p-tau217, p-tau181, GFAP, and NfL levels in cognitively healthy (CH) elderly individuals at risk for AD.

## 2. Methods

### 2.1. Study Cohort

This was a cross-sectional observational study that assessed 20 eyes from 11 cognitively normal older adults at risk for Alzheimer’s disease (age 74.9 ± 10.4 years). Individuals were predominantly Caucasian, increasing homogeneity for precise retinal measurements. All participants underwent a full ophthalmic examination by a retina specialist to exclude age-related macular degeneration, glaucoma, or other retinal degenerative diseases. Eyes with drusen, subretinal drusenoid deposits (SDDs), or structural abnormalities on OCT were excluded. Visual acuity was typically 20/20 in both eyes, and refractive error was better than −4.00 diopters, supporting the absence of clinically significant retinal pathology. The individuals had a pathological Aβ42/40 ratio based on our calculated plasma cut-off ratio of Aβ42/Aβ40 < 0.10 (mean ± SD = 0.05 ± 0.01). Based on this pathological ratio, these 11 participants were classified at the pre-AD stage. In accordance with Harrington et al. [13], participants were deemed eligible to participate through neurological and neuropsychological testing for normal cognitive function according to age, sex, and educational level. Those with a mini-mental status exam score of ≥24 were considered to have normal cognition (27.5 ± 0.7). Furthermore, participants had to have a maximum of one vascular risk factor, as one vascular risk factor is not linked to increased cerebrovascular disease in older adults. Additional demographic and clinical features are defined in Table 1.

We performed retinal imaging on all individuals and collected plasma biomarkers and MRI data with approval from the Institutional Review Board (IRB) of the University of California, Los Angeles (UCLA), with IRB #21-001404, and obtained informed consent from participants after explaining risks and benefits.

### 2.2. Retinal Layer Segmentation and Analysis

We collected retinal SD-OCT imaging using a 20 × 20 degree field (97 B-scans, Heidelberg Spectralis, Heidelberg Engineering in Heidelberg, Germany) centered on the fovea of the macula, which was then exported and transferred after de-identification to the Doheny Image Analysis Laboratory (DIAL) in Pasadena, CA, USA. Manual segmentation for retinal layers is a very time-consuming process, especially with the number of scans used. To address this challenge, we conducted automated segmentation for all the associated 10 retinal layers (11 retinal boundaries across the entire macular SD-OCT volumes) using our deep-learning-derived layer segmentation algorithm. The average for B-scan spacing was 61.65, and the average number of averaged frames was 97. The algorithm involves the calculation of pixel-to-pixel edge weights using normalized image gradients along the OCT depth axis. They are then reversed to detect dark-to-bright or bright-to-dark transitions and combined with probability maps of retinal layers generated using a fully convolutional neural network. This process decreases manual labor by over 90% while maintaining high accuracy [16,17,18]. 

After the automatic segmentation, four DIAL-trained imaging graders carefully inspected and manually corrected any errors in retinal boundary positions on each OCT B-scan using our in-house grading tool, 3D-OCTOR. The graders assessed each layer thickness across the inner retina, the outer retina, and the choroid based on the standardized Early Treatment Diabetic Retinopathy Study (ETDRS) rings (inner ring: 1–3 mm region, outer ring: 3–6 mm region). These 10 retinal layers were labeled as follows: retinal nerve fiber layer (RNFL), ganglion cell layer (GCL), inner plexiform layer (IPL), outer plexiform layer (OPL), inner nuclear layer (INL), outer nuclear layer (ONL), photoreceptor inner segment (PR-IS): external limiting membrane to ellipsoid zone, photoreceptor outer segment (PR-OS): ellipsoid zone to outer segment, retinal pigment epithelium (RPE): RPE inner to RPE outer, and choroid: Bruch’s membrane to the choroid–sclera junction (Table 2). After verifying all scans, 3D OCTOR was used to calculate the retinal layer thickness and produce ETDRS thickness maps. 

### 2.3. Plasma Biomarker Assay Methods

Using the Meso Scale Discovery (MSD) electrochemiluminescence platform (MESO QuickPlex SQ 120MM instrument) and Discovery Workbench 4.0 software (MSD, Rockville, MD, USA), we measured plasma biomarkers, including Aβ42/Aβ40, p-tau181, p-tau217, NfL, and GFAP, from blood samples collected at the Huntington Medical Research Institutes (HMRI) in Pasadena, CA, USA in accordance with clinical procedures approved by the Advarra Institutional Review Board (IRB) (HMRI # 33797). Before analysis, the blood samples were centrifuged (3000× *g*, 5 min, 20 °C) and aliquoted into 0.5 mL cryogenic-compatible tubes, then stored at −80 °C following labeling with a UID and barcode. Subjects with a plasma Aβ42/40 ratio of <0.10 (mean ± SD: 0.05 ± 0.01) were categorized with a higher risk for AD.

### 2.4. Statistical Analysis

RStudio (2024.12.0+467) was used for all statistical analyses to examine the relationship between retina layers within ETDRS-defined regions and plasma biomarkers. Ridge regression analysis was applied to model the associations between the outcomes of plasma biomarkers (Aβ42/40 ratio, p-tau181, p-tau217, GFAP, and NfL) and the entire retinal layer predictors due to potentially colinear predictors. To obtain 95% confidence intervals (CIs) for the predictor effects, we used bootstrap resampling (N = 100) at the patient level, accounting for potential correlations between eyes. Generally, the two eyes of a given participant may exhibit inter-eye correlations in retinal thickness measures and their associations with plasma biomarkers. Ideally, analyses would include a single eye per subject to ensure independence of observations. However, given the pilot nature of this study and the limited sample size, inclusion of both eyes was necessary to improve statistical power. To avoid inflating associations by treating fellow eyes as independent observations, we explicitly accounted for inter-eye correlation by performing bootstrap resampling at the patient level such that both eyes from the same participant were resampled together. This approach preserves subject-level variability while appropriately controlling for non-independence between eyes, allowing us to retain both eyes without violating statistical assumptions. We report on the estimated effects and their 95% confidence intervals. Predictors of retinal thicknesses whose 95% CIs do not include zero are considered statistically significant. We also adjusted all models for age and normalized the skewed distributions of all biomarker values through natural log transformation.

## 3. Results

**Figure 1:**
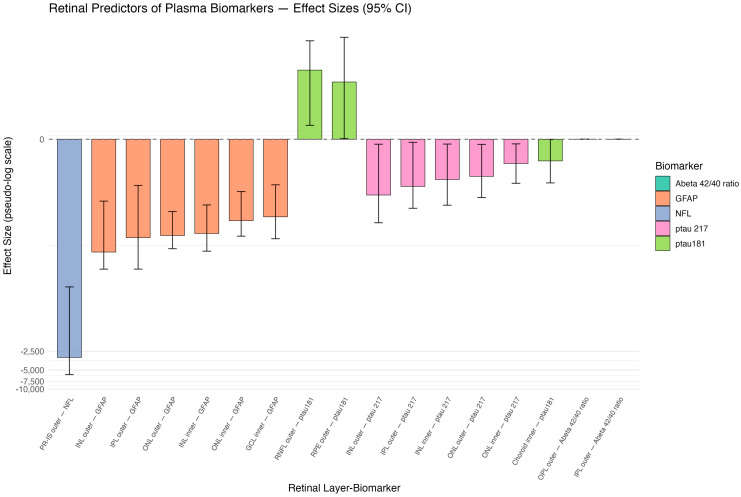
Pseudo-log-scaled effect sizes of retinal thickness predictors. Retinal layer segmentation shown by ETDRS rings: photoreceptor inner segment (IS), outer nuclear layer (ONL), inner nuclear layer (INL), outer plexiform layer (OPL), inner plexiform layer (IPL), ganglion cell layer (GCL), retinal nerve fiber layer (RNFL), retinal pigment epithelium (RPE), and choroid.

Table 2 demonstrates the ridge regression coefficient estimates with 95% confidence intervals presented in decreasing order of absolute magnitude with the raw values of plasma biomarkers. Transformed estimates in natural log were also used for the convenience of generating the illustration graph, as shown in Figure 1.

The most significant relationships of interest were determined between the retinal layer predictors and plasma biomarkers during the pre-AD stage, as shown in Table 2. Of note, in the rest of the paper, ridge regression coefficients are reported in the format of estimate followed by the 95% confidence interval in brackets, i.e., estimate [CI lower, CI upper]. The strongest association was observed between the photoreceptor inner segment (PR-IS) outer ring thickness and plasma NfL (−3126.8 [−5849.4, −231.8]), suggesting that thinning of this layer is related to increased NfL levels. There were also strong negative associations between plasma GFAP and INL outer ring (−64.1 [−120.4, −9.7]), IPL outer ring (−37.7 [−120.4, −5.3]), ONL outer ring (−34.8 [−56.7, −14.3]), INL inner ring (−32.3 [−62.1, −11.2]), ONL inner ring (−20.1 [−35.7, −6.7]), and GCL inner ring (−17.4 [−39.2, −5.2]). This indicates that the thinning of that specific retinal layer section is connected to increased GFAP levels. 

A significant positive relationship was also reported between p-tau181 (12.7 [1.1, 37.7]) and both the RNFL outer ring and RPE cell layer outer ring (8.1 [0, 42.9]). This suggests that thinner retinal layer thickness is associated with lower p-tau181 levels. There was also a negative relationship between the choroid inner ring and p-tau181 levels (−1.8 [−4.8, 0.0]), suggesting that thinning of this retinal layer is associated with increased p-tau181 levels. On the other hand, a negative relationship was observed between p-tau217 and INL outer ring (−7.7 [−21.7, −0.4]), IPL outer ring (−5.5 [−12.7, −0.2]), INL inner ring (−4.2 [−11.3, −0.4]), ONL outer ring (−3.7 [−8.5, −0.4]), and ONL inner ring (−2.0 [−4.9, −0.3]). This suggests that thinning of these specific retinal layer section thicknesses and higher p-tau217 plasma levels are related. The Aβ42/40 ratio was also found to have no significant association with the OPL outer ring and IPL outer ring due to lack of variance or collinearity.

## 4. Discussion and Conclusions

In our pilot study, we analyzed the relationship between the various retinal layers and plasma biomarkers, specifically NfL, GFL, p-tau181, p-tau217, and the Aβ42/40 ratio. These associations reveal early processes connecting retina and brain pathology in “pre-AD” patients. Our analytical approach shows the size effects of each retinal layer at a cross-section of the pre-AD timeline when cognition is still normal. We observe that the outer retinal layers, particularly the photoreceptor inner segment (PR-IS), show early vulnerability to neuroaxonal injury, as reflected by the strong negative association between PR-IS thinning and elevated plasma NfL levels, exhibiting greater structural involvement compared with the inner retinal layers.

We found several significant associations, with the most significant being between NfL and the PR-IS outer ring. Photoreceptor inner segments are highly metabolically active structures and are particularly sensitive to IL-1β-mediated inflammatory signaling. Experimental studies have shown that CX3CR1- and P2RX7-dependent microglial activation drives IL-1β secretion, which in turn promotes inflammation-associated photoreceptor degeneration. Moreover, IL-1β upregulation has been linked to increased expression and release of neurofilament light (NfL) due to its role in cytoskeletal breakdown during neuroaxonal injury. These mechanisms provide biological support for the strong association observed between PR-IS thinning and elevated plasma NfL levels in our cohort. NfL is a blood-based biomarker for neuroaxonal damage and has been used to predict cognitive decline and brain atrophy [19]. Recent studies have focused on the relationship between NfL levels and inner retinal layers such as the ganglion cell layer and inner plexiform layer [19]. Few studies have focused on the relationship between outer photoreceptor thickness and plasma NfL; thus, our novel finding should be attempted to be replicated. In AD mice, photoreceptor cells have shown symptoms of degeneration [20]. The PR-IS outer ring thinning with increased NfL levels may reflect mitochondrial dysfunction and decreased choroidal supply, which is common in Alzheimer’s [21]. Increased plasma NfL and PR-IS thinning could be due to a shared consequence of this oxidative stress during a stage where visual acuity and cognition are still intact. Studies have shown that an increase in plasma GFAP and NfL levels occurs during the transition from healthy aging to pre-AD stages before other AD biomarkers are observed [2]. NfL has been shown to increase at a later change point, indicating a higher AD risk within 9 years of diagnosis [2,22]. It has also demonstrated relatively high accuracy in predicting the transition from mild cognitive impairment (MCI) to Alzheimer’s dementia [23]. Our findings that neurodegeneration likely affects the outer retinal layers more than the inner layers are further supported by a recent study by Kim et al. [24], who also showed similar results in amyloid-PET-positive CH elderly subjects compared to amyloid-PET-negative ones.

We observed negative associations between GFAP and a number of retinal layers: INL outer ring, IPL outer ring, ONL outer ring, INL inner ring, ONL inner ring, and GCL inner ring. GFAP is an astrocytic cytoskeletal protein that indicates astrogliosis, which has been linked to early AD [25]. Thinning of these retinal layers could reflect glial loss or Muller glia dysfunction, which could increase astrocyte activation, thus increasing GFAP levels [26]. Specifically, the GCL inner ring thinning corresponds to the foveal region, where there is a significant increase in the ratio of astrocytes to retinal ganglion cells in AD compared to healthy patients [27]. Blanks et al.’s study also demonstrated an increase in GFAP immunoreactivity in astrocytes and Muller cell end-feet in this region [27]. However, GFAP is a systemic marker, revealing astroglial reactions in other parts of the body. Thus, the relationships found in our study are suggestive but not direct proof for local retinal gliosis.

We also observed positive relationships between p-tau181 and both the RNFL outer ring and RPE outer ring. Previous studies have shown that choriocapillaris flow blockage can significantly affect RPE thickening [28]. Additionally, a negative association was found between p-tau181 and the choroid inner ring. The choroid provides the outer retina’s blood supply, so thinning of this layer can lead to hypoperfusion that can cause outer retinal damage or metabolic stress. Results from Asanad et al. have shown that Aβ deposition and local inflammation occur in the choroid retinal layer, leading to thinning [29]. This could lead to a cascade of accumulation in plaques, activating microglia and astrocytes, releasing plasma p-tau181 [30]. Phosphorylated p-tau181 in the pre-AD stages could represent a transition to the neurodegeneration stage rather than the synaptic dysfunction of the T+ stage.

P-tau217 was observed to be negatively associated with several inner and outer retinal layers, including the INL and IPL outer rings, the INL inner ring, and the ONL inner and outer rings. p-tau217 is one of the most specific biomarkers for AD, relating to trans-synaptic dysfunction and damage [31]. We determined stronger associations in the outer ETDRS ring than in the inner ring regions, which was clearly evident in the PR-IS, RNFL, INL, IPL, ONL, and OPL. This suggests that the peripheral retina region could be more vulnerable to early AD processes. We observed that increased plasma GFAP levels correlated most strongly with thinning in the outer ring of INL, OPL, and ONL. Likewise, we observed a strong association between the plasma p-tau217 and the outer ring INL, IPL, and ONL. These correlations suggest that the outer retinal regions are particularly informative for detecting early AD-related changes. This region could also provide clearer biomarkers for AD, as the central retina could be confounded by patterns from other diseases, such as age-related macular degeneration.

On histopathology, p-tau217 and p-tau181 have been observed to accumulate in the soma and dendrites of amacrine and horizontal cells in the INL of AD retinas. Based on histological sections representing different stages along the tau trajectory in AD retinas, p-tau217 and p-tau181 deposits were identified in the OPL, IPL, and INL [32,33].

We considered whether individual participants exhibited concurrent alterations across multiple retinal layers and whether integrating multiple plasma biomarkers enhances the detection of preclinical Alzheimer’s disease. In this study, all potentially affected retinal layers across the entire retina were evaluated simultaneously using a ridge regression framework with patient-level bootstrapping, allowing us to assess multilayer retinal associations within the same individual. Indeed, several participants demonstrated thickness changes across more than one retinal layer in association with plasma biomarker levels, consistent with the multifactorial and spatially heterogeneous nature of early neurodegenerative processes in preclinical AD.

In addition, the use of multiple plasma biomarkers is expected to improve sensitivity and specificity for detecting preclinical disease. Each biomarker interrogates a distinct pathological pathway, including amyloid dysregulation (Aβ42/40), tau phosphorylation (p-tau181 and p-tau217), astroglial activation (GFAP), and neuroaxonal injury (NfL). The complementary nature of these biomarkers provides a more comprehensive biological profile than reliance on any single analyte and may improve the ability to characterize an individual’s position along the AD continuum. Although formal sensitivity and specificity analyses were beyond the scope of this pilot study, our findings support the value of a multilayer retinal and multi-biomarker approach for early AD detection.

This study had several limitations due to being a pilot study. Notably, the sample size was small, which limits statistical precision, as seen by the broad confidence intervals. Our results must be carefully interpreted because of how large the confidence intervals are and could be sensitive to outliers. Second, because this was a cross-sectional study, we are unable to detect temporal causality. Longitudinal follow-up would be needed to determine if retinal thinning precedes or follows the biomarker changes. Third, although OCT segmentation of retinal layers is relatively precise, there can still be segmentation errors. Lastly, although our results suggest that retinal thickness features may serve as promising predictors for pre-AD, further research is needed to determine whether these significant predictors can reliably distinguish pre-AD individuals from biomarker-negative, cognitively normal control individuals. Because our cohort was intentionally restricted to individuals with abnormal plasma biomarkers consistent with pre-AD, we were unable to directly compare retinal layer thicknesses with normative aging patterns. Because only subjects with abnormal findings were included and no control group was used, our results describe associations within abnormal cases only and were not intended to directly address differences between normal and abnormal populations. Our analysis, therefore, focused on continuous associations within the pre-AD range. We acknowledge the importance of including age-matched biomarker-negative controls, and future work will incorporate such a comparison group to establish normative baselines and strengthen interpretability.

Despite these limitations, our results still provide useful estimates for larger prospective studies. Using a novel retinal segmental algorithm allows for efficient segmentation for future larger cohorts. This technique accurately measures the photoreceptor outer segment and excludes subretinal drusenoid deposits (SDDs) while also delineating the RPE, excluding drusen [34,35,36]. Although ridge regression helped mitigate collinearity among the retinal layer thickness predictors, the findings should be considered hypothesis-generating rather than confirmatory.

In this pilot study, we determined associations between several retinal layer thicknesses and plasma AD biomarkers. PR-IS thinning stood out significantly as an indicator of neurodegeneration. Although statistical precision was limited, these findings support the potential for retinal imaging to be a non-invasive way for early biomarker detection for pre-AD patients. Studies have shown that plasma AD biomarkers can improve the prediction of cognitive decline in CH elderly patients [37,38]. This emphasizes the need to confirm our retinal imaging findings with plasma AD biomarkers. Future studies can utilize larger study cohorts to increase the statistical precision of our associations found in this study. An artificial intelligence model could be developed to determine individuals at increased risk of developing dementia through retinal imaging and plasma biomarkers, allowing for early neurological evaluations. 

## Figures and Tables

**Table 1:** Demographic and clinical characteristics of the study cohort. Continuous variables are shown as mean ± SD, and categorical variables are shown with percentages.

Characteristic	N	Mean ± SD	Percent	Notes
Total number of subjects	11			
Total number of eyes	20			
Age (years)	11	74.9 ± 10.4		
Females	9		82%	
ApoE ε 3/3	9		82%	
ApoE ε 3/4	2		18%	
Plasma Aβ42/40 ratio	11	0.05 ± 0.01		<0.10 = increased risk of AD
NFL	11	178,722.20 ± 88,823.43		
GFAP	11	59,253.03 ± 23,691.11		
ptau217	11	7689.32 ± 2955.07		
ptau181	11	2928.47± 2346.68		
Mini-Mental Status Exam score	11	26.0 ± 0.9		≥24 = normal cognition
Best-corrected visual acuity	20	20/25 ± 2.2		

**Table 2:** Ridge regression analysis of retinal layer thicknesses and plasma biomarkers in decreasing order of absolute magnitude.

Predictor	Outcome	Estimate (95% CI)	Natural Log Estimate	CI Width	Direction	Significance
PR-IS outer ring	NfL	−3126.8 (−5849.4, −231.8)	−8.0	5617.6	Negative	Yes
INL outer ring	GFAP	−64.1 (−120.4, −9.7)	−4.2	110.7	Negative	Yes
IPL outer ring	GFAP	−37.7 (−120.4, −5.3)	−3.6	115.1	Negative	Yes
ONL outer ring	GFAP	−34.8 (−56.7, −14.3)	−3.5	42.4	Negative	Yes
INL inner ring	GFAP	−32.3 (−62.1, −11.2)	−3.5	50.9	Negative	Yes
ONL inner ring	GFAP	−20.1 (−35.7, −6.7)	−3.0	29.0	Negative	Yes
GCL inner ring	GFAP	−17.4 (−39.2, −5.2)	−2.9	34.0	Negative	Yes
RNFL outer ring	p-tau181	12.7 (1.1, 37.7)	2.5	36.6	Positive	Yes
RPE cell layer outer ring	p-tau181	8.1 (0, 42.9)	2.1	42.9	Positive	No
INL outer ring	p-tau217	−7.7 (−21.7, −0.4)	−2.0	21.3	Negative	Yes
IPL outer ring	p-tau217	−5.5 (−12.7, −0.2)	−1.7	12.5	Negative	Yes
INL inner ring	p-tau217	−4.2 (−11.3, −0.4)	−1.4	10.9	Negative	Yes
ONL outer ring	p-tau217	−3.7 (−8.5, −0.4)	−1.3	8.1	Negative	Yes
ONL inner ring	p-tau217	−2.1 (−4.9, −0.3)	−0.7	4.6	Negative	Yes
Choroid inner ring	p-tau181	−1.8 (−4.8, 0)	−0.6	4.8	Negative	No
OPL outer ring	Aβ42/40 ratio	0 (0, 0)	0.0	0.0	Positive	No
IPL outer ring	Aβ42/40 ratio	0 (0, 0)	0.0	0.0	Positive	No

## Data Availability

The datasets used and/or analyzed in the current study are available from the corresponding authors on reasonable request.
